# Alcohol’s Effects on Gene Expression

**Published:** 1995

**Authors:** Michael F. Miles

**Affiliations:** Michael F. Miles, M.D., Ph.D., is an associate professor of neurology at the Ernest Gallo Clinic and Research Center, University of California, San Francisco, School of Medicine, San Francisco, California

**Keywords:** AODE (alcohol and other drug effects), expression, AOD dependence, AOD tolerance, genetic mapping, DNA, RNA, proteins, enzymes, biochemical mechanism

## Abstract

Altering the expression of specific genes in the brain is one of the mechanisms through which the organism may adapt to chronic alcohol exposure. Several molecular biology methods allow researchers to isolate alcohol-regulated genes from cultured cells or laboratory animals. These techniques have led to the identification of numerous genes whose expression is increased or decreased in cells that have been exposed to alcohol. The protein products of these genes serve a wide variety of functions in normal cells, such as the processing of protein molecules or signal transmission within cells. Preliminary studies are providing insights into the mechanisms through which alcohol may alter the expression of these genes.

Chronic heavy drinkers can tolerate alcohol without exhibiting obvious signs of impairment at doses that in other people would be incapacitating or even fatal. For example, in nonalcoholic people, blood alcohol concentrations (BAC’s) of 0.6 percent and more usually cause death from respiratory failure. Chronic heavy drinkers, in contrast, frequently survive similar alcohol doses and may even appear sober (Charness et al. 1989). This acquired resistance to the effects of high alcohol doses is called tolerance and is one of the criteria used to establish a medical diagnosis of alcohol dependence (American Psychiatric Association 1994).

The development of tolerance indicates that the brain has adapted to the chronic presence of alcohol. As a consequence of this adaptation, the brains of many alcholics need elevated alcohol levels to function normally. Conversely, drinking cessation leads to adverse effects in the form of withdrawal symptoms. This state is known as physical dependence.

The mechanisms underlying the phenomena of tolerance and dependence are currently unknown. Increased metabolism of alcohol does not explain why alcoholics’ brains function differently from those of nontolerant people in the presence of equal alcohol concentrations. The time course for the development of tolerance and dependence does, however, suggest a general hypothesis: Changes in the expression of specific genes may account for at least some of the dramatic changes evident in chronic heavy drinkers’ brain functions.

Behavioral studies in both humans and animals have found that tolerance and physical dependence can develop over several days of chronic alcohol exposure. Such a timeframe is consistent with the duration required to induce changes in gene expression in response to alcohol exposure. The term “changes in gene expression” refers to increases or decreases in the amount of gene products (i.e., proteins) synthesized from specific genes.^1^ Other regulatory events in the cells, such as activating or deactivating proteins by adding or deleting phosphate groups, occur much more rapidly, that is, within minutes. Conversely, some changes that have been observed after chronic alcohol exposure, such as the “rewiring” of nerve cell connections or decreases in the number of brain cells, occur too slowly to explain the full range of events seen with tolerance and dependence in alcoholics.

Over the past few years, a number of researchers have identified several genes whose expression is modified after exposure of animals or of cells in tissue culture to alcohol. For some of these genes, expression is increased (Charness et al. 1988; Gayer et al. 1991; Miles et al. 1991), whereas for other genes, expression is decreased (Mochly-Rosen et al. 1988; Morrow et al. 1990). This article briefly describes the identification of alcohol-responsive genes and their possible physiological roles in the body’s response to alcohol. Finally, this article presents possible mechanisms through which alcohol may regulate gene expression.

## Identifying Alcohol-Regulated Genes

To identify genes whose expression in the brain is altered after alcohol exposure, researchers employ several strategies. As starting material, they generally use nerve cells grown and incubated with alcohol in tissue culture or tissue from animals exposed to alcohol. Once a model system is chosen, different technical approaches can be used to characterize the alcohol-responsive genes. Two such approaches are briefly described below.

### Study of Candidate Genes

The most straightforward approach to identifying alcohol-regulated genes is to study genes that already have been isolated and that are known or suspected to contribute to alcohol’s effects on the body. Such genes are called candidate genes. By comparing protein production of the candidate genes in cells that have or have not been exposed to alcohol, researchers can determine whether alcohol increases, decreases, or has no effect on the genes’ expression.

An example of this strategy is the study of the gene that encodes the enzyme tyrosine hydroxylase (TH). TH catalyzes the first step in the synthesis of a group of neurotransmitters^2^ called catecholamines, which includes dopamine and norepinephrine. Alcohol affects the levels of some catecholamines; this process may contribute to the changes in brain function observed after chronic alcohol exposure. To analyze whether alcohol alters the expression of TH, Gayer and colleagues (1991) treated neural cells with alcohol levels corresponding to BAC’s observed in alcoholics. After 3 days, the amounts of both TH messenger RNA (mRNA), an intermediary molecule synthesized during the conversion of genetic information into a protein, and TH protein in the alcohol-treated cells increased almost twofold over the untreated control cells.

### Subtractive RNA Hybridization

**Figure 1:**
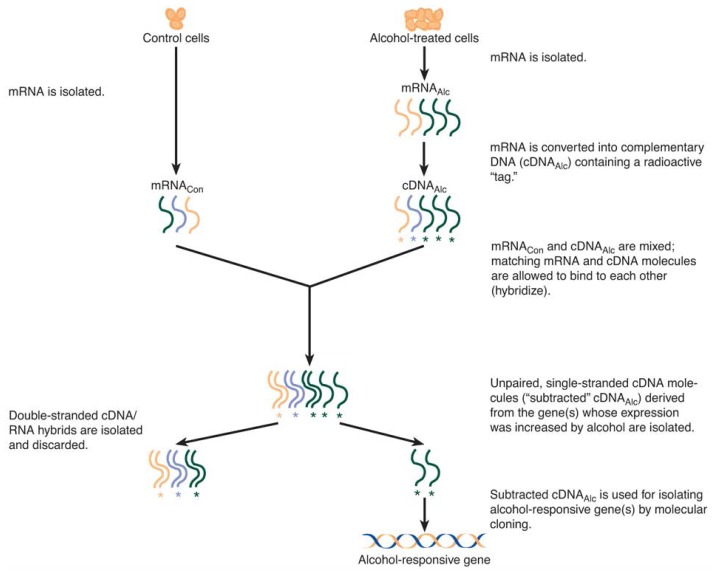
Outline of the subtractive RNA hybridization process to isolate and identify genes whose expression is increased by alcohol. The technique uses cultured cells (e.g., neural cells) grown in the presence or absence of alcohol.

To discover new genes that may be regulated by alcohol, researchers must use more sophisticated strategies. One method for identifying unknown genes whose expression is increased by alcohol is subtractive RNA hybridization (figure 1). This technique uses the mRNA from two batches of neural cells. The RNA from each batch of cells is a mixture, or pool, of mRNA’s from many different genes. If both batches of cells are grown under the same conditions, both pools should contain similar amounts of each individual mRNA. If one batch of cells is grown in the presence of alcohol, and if alcohol increases the expression of certain genes, then the RNA pool from the alcohol-treated cells should contain higher levels of the RNA’s from the alcohol-induced genes than the RNA pool derived from the untreated cells.

To identify the alcohol-induced mRNA’s, researchers first copy the RNA pool from the alcohol-treated cells into DNA molecules, which are more stable and easier to handle in the experiment. During this process, the DNA molecules also are labeled with a radioactive “tag” to distinguish them from the mRNA’s of untreated cells. Next, both the labeled DNA from the alcohol-treated cells and the RNA pool from the untreated cells are mixed with each other in specific proportions. Under certain conditions, DNA pieces from the alcohol-treated cells form pairs with corresponding RNA pieces from the untreated cells. This process is called hybridization. However, because there is an excess of DNA pieces derived from the alcohol-induced mRNA’s, not every one of these pieces will be able to find a partner among the mRNA’s derived from untreated cells. Because they are “single-stranded,” these unpaired DNA pieces can be biochemically separated from the DNA–RNA hybrids in the mixture. Due to their radioactive tags, these single-stranded DNA molecules then can be used to identify the alcohol-regulated genes from which they originated.

**Figure 2:**
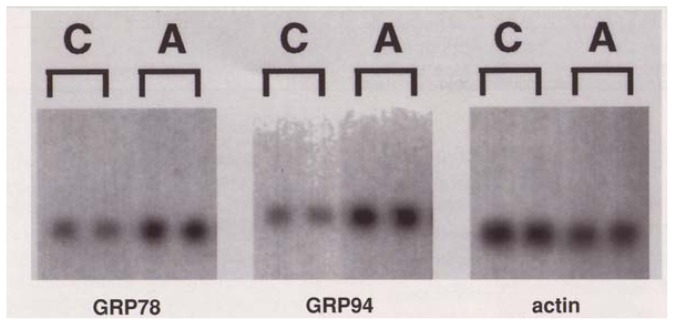
Alcohol increases the expression of two genes encoding the molecular chaperone proteins GRP78 and GRP94 but not of the gene encoding the protein actin. To assess the effect of alcohol on gene expression, mRNA’s isolated from alcohol-treated cells (A) and untreated control cells (C) were incubated with radioactive probes that bind to the specific mRNA’s and which can be detected on x-ray film. The black bands give a visual estimate of the relative amount of the particular mRNA. Alcohol treatment increased the amount of GRP78 and GRP94 mRNA but had no significant effect on the amount of actin mRNA in the cells.

Using a similar approach, Miles and colleagues (1994) isolated DNA pieces representing several genes whose expression was increased in the presence of alcohol. When the researchers compared these genes to data on other genes stored in a computer database, they found that some of the genes already had been identified in unrelated experiments. For example, two DNA pieces contained the genes for so-called molecular chaperones (discussed in more detail below). Alcohol treatment of neural cell cultures significantly increased the mRNA amounts of these two molecular chaperones, GRP78 and GRP94 (figure 2). In contrast, other alcohol-regulated genes isolated by subtractive hybridization had not been described before.

The finding that seemingly unrelated genes are regulated by exposure of the cells to alcohol sheds new light on how alcohol affects known functions of nerve cells. The identification of unknown alcohol-responsive genes enables scientists to discover additional effects that alcohol has on both individual cells and the whole organism.

## The Physiological Roles of Alcohol-Regulated Genes

The alcohol-regulated genes and corresponding protein products that have been identified to date are involved in a wide variety of cell functions. Although a comprehensive review of all these proteins is beyond the scope of this article, some examples will be described here in more detail. Table 1 summarizes the functions of additional alcohol-regulated genes.

### Tyrosine Hydroxylase

As mentioned earlier, the enzyme TH catalyzes a key reaction in the synthesis of a group of neurotransmitters called catecholamines. One of the catecholamines, dopamine, is involved in regulating motor functions, cognitive functions, emotions, and aggression. Studies in rats found that when the animals received an alcohol dose, the release of dopamine in certain brain areas increased (Di-Chiara and Imperato 1985). Findings such as these could explain why dopamine has been implicated in the sensation of reward associated with abused drugs (Koob 1992), including alcohol, and in the development of tolerance to alcohol (Ritzmann and Tabakoff 1976).

Because TH is essential for the first step of dopamine synthesis, alcohol-induced changes in TH gene expression could contribute to the increased dopamine levels observed after alcohol administration. As described above, experiments with cultured neural cells found that both TH mRNA and protein levels are elevated after prolonged treatment of the cells with alcohol (Gayer et al. 1991). These findings indicate one mechanism through which alcohol could affect neurotransmitter levels and thus contribute to tolerance development in chronic heavy drinkers. Animal studies on other drugs of abuse (e.g., cocaine and amphetamines) also found increased TH expression in brain regions associated with reward sensations (Breitner-Johnson and Nestler 1991).

Researchers now are studying the molecular mechanisms through which alcohol may regulate TH gene expression. The implications of such studies may have significance beyond the role of the TH gene itself in brain functioning, because the same mechanisms also may apply to many other genes. (See the section “Mechanisms of Alcohol-Dependent Regulation of Gene Expression,” below.)

### Molecular Chaperones

Some of the genes whose mRNA increases after alcohol treatment belong to a family of genes that code for proteins called molecular chaperones. One function of molecular chaperones is, as the name implies, to escort newly made proteins from the point where they are synthesized to their final destination in the cells (Ellis 1994). The chaperone is vital for the correct functioning of proteins.

Most proteins, after their initial synthesis, undergo chemical modifications that are essential if the proteins are to reach their destinations in or outside the cell and to function properly. The modifications, which usually consist of the addition of sugar molecules or phosphate molecules, occur in specific structures, or organelles, within the cell. Incorrectly modified proteins are degraded immediately or may not function properly if they reach their final destination. The molecular chaperones associate with newly synthesized proteins, guide them to the appropriate organelles, and ensure that the proteins achieve and maintain their correct shapes and modifications (High-tower 1991). Molecular chaperones also are required to transport (i.e., uptake) certain proteins, such as receptors for neurotransmitters or hormones, from the cell surface into the cells (Chappell et al. 1986).

Research has shown that alcohol interferes with the normal transport of proteins through the cell. For example, Tuma and colleagues (1991) found that alcohol inhibited the uptake of specific receptors from the cell surface into liver cells. This alteration in the trafficking of receptor proteins could alter liver-cell function. Increased production of chaperone proteins after extended alcohol exposure, however, may compensate for these changes by altering the transport and modification of other proteins. This process could represent a way for cells to adapt to the continued presence of alcohol (i.e., develop tolerance). Similarly, acute exposure of the nervous system to alcohol alters the function of several proteins on the surface of nerve cells that serve as receptors for neurotransmitters. Molecular chaperone-induced changes in the trafficking and processing of neurotransmitter receptors might enable the brain to adapt to the presence of alcohol.

### GTP-Binding Proteins

GTP (guanosine triphosphate)-binding proteins (G proteins) are part of the signaling cascade that transmits extracellular signals (e.g., hormone signals) into and within the cell. Through several steps, this process leads to changes in the expression and function of specific genes and proteins in response to the incoming signal. Acute doses of alcohol strongly increase certain cellular responses to incoming signals, including the activity of G proteins, a reaction that may be harmful to the cell (Gordon et al. 1992).

Cells contain two major kinds of G proteins. Stimulatory G protein (G_s_ protein) activates the next enzyme in the signal transmission cascade; inhibitory G protein (G_i_ protein) inhibits the next enzyme. Studies in neural cells found that when the cells were exposed to alcohol for several days, G_s_ levels decreased and G_i_ levels increased (Charness et al. 1988; Mochly-Rosen et al. 1988). Such a modification in G-protein expression could serve to diminish the cell’s response to incoming signals. As a result, the cells would become less sensitive to a variety of stimuli, thus avoiding the “overreaction” seen in the presence of an acute alcohol dose.

## Mechanisms of Alcohol-Dependent Regulation of Gene Expression

Researchers have learned much about alcohol’s effects on the body by identifying genes whose expression is modified by alcohol. Not much is known, however, about the way in which alcohol exerts these effects. Current research efforts are focusing on detailed studies of the structure and function of individual genes to determine the sites and mechanisms of alcohol’s action.

### Alcohol’s Effects on Promoters and Transcription Factors

Much of the research on how alcohol affects gene expression focuses on DNA regions adjacent to the genes being regulated, the so-called promoters. Promoters function like switches that determine when, and how strongly, a gene is expressed. These switches are operated by the binding of certain proteins, the transcription factors, to specific stretches of DNA within the promoter region. (For more information on promoters and transcription factors, see sidebar, p. 241.)

**Promoters and Transcription Factors**

Every cell in an organism contains the complete genetic information needed to create that organism. Not all cells, however, express all those genes all the time. For example, liver cells do not need to synthesize the neurotransmitters that relay signals among nerve cells. Similarly, brain cells have no use for liver-specific enzymes. And even a brain cell does not produce neurotransmitters continuously. These examples illustrate the requirement for a specific pattern of gene expression in each individual cell. An imbalance in the patterns—for example, the expression of a gene in the wrong cell, at the wrong time, or in the wrong amount—can have disastrous consequences for the organism. Conversely, gene expression patterns also must be flexible so that they can be adjusted rapidly to the cell’s changing requirements in response to internal processes or environmental influences.

To ensure the coordinated expression of genes at the right time and in the right tissues of an organism, regulatory elements have evolved that enable a cell to turn the transcription of each gene on and off, as needed. These “switches,” or promoters, are stretches of DNA directly adjacent to the gene sequence itself. Some promoters represent on/off switches, regulating gene expression by an all-or-nothing pattern. Other promoters resemble “dimmers,” which allow for gradual changes in the level of gene expression depending on the environmental conditions and the requirements of the cell at any given moment.

In addition to controlling the level of gene expression (i.e., the level of transcription), promoters also determine the site where transcription begins. The enzyme that performs the chemical reactions of transcription recognizes a certain nucleotide sequence in the promoter as the transcription initiation site and binds to that site to begin its work.

Promoters regulate gene expression by providing binding sites for certain proteins, the so-called transcription factors (see figure). Many different transcription factors exist in the cells, and researchers constantly are discovering new ones. The binding of certain transcription factors to the promoter can activate gene expression by facilitating the access of transcription enzymes to the gene. The binding of other transcription factors to the promoter, however, inhibits transcription by blocking the transcription enzyme from accessing the gene. Some transcription factors are present in the cells at all times; others are synthesized only at specific times during development or under specific environmental conditions. All promoters contain binding sites for several transcription factors, so that the level of gene expression can be finely tuned through coordinated variation in the kind and abundance of the factors present in the cell at a particular time.

**Figure:**
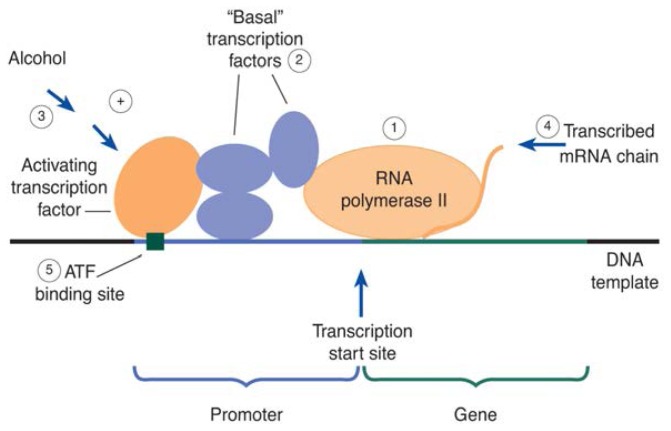
Regulation of gene expression through promoters and transcription factors. Adjacent to genes are promoters, DNA regions that control how much RNA is transcribed from that gene. (1) The enzyme RNA polymerase II initiates transcription at a specific site in the promoter. (2) Certain “basal” transcription factors control the binding of RNA polymerase to this site. Other proteins that bind to specific short DNA stretches in the promoter and basal factors work together to activate or inhibit transcription. Drugs such as alcohol may modify the activity of those factors. (3) Here, alcohol is arbitrarily assumed to increase the activity of an activating transcription factor (ATF), resulting in (4) increased RNA synthesis from the alcohol-responsive gene. (5) Genes lacking this particular ATF binding site would not respond to alcohol.

Each transcription factor has a specific sequence of DNA nucleotides that it recognizes in the promoter and to which it binds. For many factors, these sequences are known and have been studied in detail. Researchers have introduced changes (i.e., mutations) into these sequences to study how the mutations affect binding and activity of a transcription factor. In other approaches, pieces of DNA containing the transcription factor binding sites are removed from the promoter to study how these deletions—and the resulting inactivation of specific transcription factors—affect expression of a particular gene. Finally, by analyzing the nucleotide sequence of the promoter region of a new gene, researchers frequently can predict which transcription factors are involved in regulating the gene’s expression and can make educated guesses about the effects of certain manipulations of the cell or its environment on gene expression.

— Michael F. Miles and Susanne Hiller-Sturmhöfel

To study whether alcohol affects gene expression by interfering with the functions of transcription factors, researchers removed various sections of the promoters of alcohol-dependent genes and then examined whether gene expression was still sensitive to alcohol. For example, the promoters of both the TH gene and an alcohol-dependent molecular chaperone, Hsc70, contain short DNA stretches that, if placed in the promoter regions of genes unresponsive to alcohol, can confer alcohol-responsiveness to these genes. Preliminary studies by Miles and colleagues (1993) found that removing these DNA sequences from the Hsc70 promoter almost completely abolished alcohol’s ability to affect Hsc70 expression. Experiments are in progress to identify the transcription factors that bind to the alcohol-responsive promoter regions.

### Alcohol’s Effects on Signal Transmission Cascades

As mentioned earlier, alcohol affects the activity of G proteins, which play an important role in the signal transmission within cells. G proteins regulate a subsequent step in the signal transmission cascade, the generation of so-called “second messengers.” These messengers generally are small molecules that relay signals by modulating the activity of other signaling molecules and by altering the expression of various genes. One second messenger, cyclic adenosine monophosphate (cAMP), controls the expression of a wide variety of genes, including TH (Roesler et al. 1988).

Previous studies have found that acute alcohol treatment of cells leads to an increase in cAMP levels; chronic alcohol treatment decreases the level of cAMP in cells. Accordingly, alcohol-induced changes in cAMP levels could alter the expression of many cAMP-dependent genes, including TH. So far, however, researchers have not been able to determine unequivocally whether alcohol affects TH gene expression through changes in the cAMP concentration or through other mechanisms.

Another component of a signaling cascade is protein kinase C (PKC), an enzyme that regulates the function of various proteins by adding phosphate groups to them. PKC also is known to regulate the expression of certain genes. Messing and colleagues (1991) found that the activity of PKC increased in neural cells chronically exposed to alcohol. Thus, alcohol-induced changes in PKC activity could alter the expression of other genes.

## Future Directions

A growing number of studies using neural cell cultures or brain cells from animals exposed to alcohol have documented alcohol-induced changes in gene expression. The exact roles that these genes play in the adaptation of intact animals to long-term alcohol exposure, however, remain to be determined. Alcohol-responsiveness in intact animals still must be confirmed for many genes that respond to alcohol in cell cultures (see table 1).

Investigators also are initiating innovative approaches, such as transgenic animals, in which the expression of a specific gene has been either increased or completely shut off, to study the roles of individual genes in the physiological and behavioral consequences of acute and chronic alcohol use (Harris et al. 1995).^3^ If researchers are able to identify individual genes linked to tolerance, these findings may have important consequences. By studying the function and mechanisms involved in the regulation of these genes, researchers may someday generate new treatments for alcoholism. A drug that interferes with alcohol-dependent regulation of gene expression, for example, might aid other treatments attempting to reduce the long-term alcohol-seeking behavior associated with alcoholism.

## Figures and Tables

**Table 1:** Summary of Alcohol-Responsive Genes Identified in Cell Cultures or Animal Models and From Human Alcoholics^1^

	Tissue or Cell Type Studied	mRNA Abundance After Alcohol	Function
**Neurotransmitters or Hormones**			
α2–adrenergic receptor	Neural cells	Increased	Binding of chemical messengers (e.g., neurotransmitters or hormones) and signal transmission into the cells
Gamma-aminobutyric acid (GABA) receptor subunits	Rat brain	Increased or decreased^2^	
δ–opioid receptor	Neural cells	Increased	
Retinoic acid receptor	Mouse liver	Increased	
Thyroid hormone receptor	Mouse liver	Increased	
Preproenkephalin	Rat brain	Increased	Neurotransmitters and/or hormones
Vasopressin	Mouse brain	Decreased	
β–luteinizing hormone	Rat brain	Decreased	
Tyrosine hydroxylase	Neural cells	Increased	Synthesis of catecholamine neurotransmitters

**Signaling Proteins**			
G_α__s_	Rat brain, neural cells	Decreased	Proteins involved in signal transmission within cells
G_α__i_	Rat brain neural cells, human lymphocytes		
Phosducin-like protein (PhLP)	Neural cells	Increased	

**Molecular Chaperones**			
GRP78	Neural cells	Increased	Modification and transport of proteins within and into cells
GRP94			
Hsc70			
Signal peptidase	Rat brain	Increased	Processing of proteins that are secreted or located within the cell membrane

**Others**			
a_1_–protease inhibitor	Human liver tumor	Increased	Released by certain cells in response to tissue inflammation
Tumor necrosis factor	Human leukemia cells	Increased	
c-jun proto-oncogene	Human keratinocytes	Increased	Transcription factor
Glial fibrillary acidic protein (GFAP)	Rat brain	Increased	Component of the internal skeleton of certain brain cells
Glucose transporter-1	Fetal rat brain	Decreased	Glucose transport
Pro α1(I) collagen	Human liver	Increased	Connective tissue protein
Albumin	Human liver	Increased	Major blood protein
Low-density lipoprotein receptor	Rat liver	Increased	Cholesterol metabolism
HMG coenzyme A			
Apolipoprotein E			

1 Original literature citations for these studies are available by request from the author.

2 Alcohol can either increase or decrease GABA receptor subunits, depending on the particular subunit and brain region studied.

## Footnotes

1 For a brief description of the processes involved in converting genetic information from DNA into a protein product, see the article by Hiller-Sturmhöfel et al., pp. 206–213.

2 For a definition of this and other technical terms used in this article, see central glossary, pp. 182–183.

3 For more information on the use of transgenic animals in alcohol research, see the article by Hiller-Sturmhöfel et al., pp. 206–213.
